# Two Novel Pathogenic *FBN1* Variations and Their Phenotypic Relationship of Marfan Syndrome

**DOI:** 10.1055/s-0040-1714092

**Published:** 2020-08-20

**Authors:** Sinem Yalcintepe, Selma Demir, Emine Ikbal Atli, Murat Deveci, Engin Atli, Hakan Gurkan

**Affiliations:** 1 Department of Medical Genetics, Faculty of Medicine, Trakya University, Edirne, Turkey; 2 Department of Pediatric Cardiology, Faculty of Medicine, Trakya University, Edirne, Turkey

**Keywords:** Marfan syndrome, novel variation, next-generation sequencing

## Abstract

Marfan syndrome is an autosomal dominant disease affecting connective tissue involving the ocular, skeletal systems with a prevalence of 1/5,000 to 1/10,000 cases. Especially cardiovascular system disorders (aortic root dilatation and enlargement of the pulmonary artery) may be life-threatening. We report here the genetic analysis results of three unrelated cases clinically diagnosed as Marfan syndrome. Deoxyribonucleic acid (DNA) was isolated from EDTA (ethylenediaminetetraacetic acid)-blood samples of the patients. A next-generation sequencing panel containing 15 genes including *FBN1* was used to determine the underlying pathogenic variants of Marfan syndrome. Three different variations, NM_000138.4( *FBN1* ):c.229G > A(p.Gly77Arg), NM_000138.4( *FBN1* ):c.165–2A > G (novel), NM_000138.4( *FBN1* ):c.399delC (p.Cys134ValfsTer8) (novel) were determined in our three cases referred with a prediagnosis of Marfan syndrome. Our study has confirmed the utility of molecular testing in Marfan syndrome to support clinical diagnosis. With an accurate diagnosis and genetic counseling for prognosis of patients and family testing, the prenatal diagnosis will be possible.

## Introduction

Marfan syndrome (MFS) (Online Mendelian Inheritance in Man or OMIM#154700) is an autosomal dominant inherited connective tissue disorder affecting cardiovascular, ocular, skeletal systems with a prevalence of one of 5,000 to one of 10,000 cases. 1 Life-threatening cardiovascular system findings include aortic root aneurysm, mitral valve prolapse with/without regurgitation, tricuspid valve prolapse, enlargement of the proximal pulmonary artery, and dilatation of the aorta. Ocular findings are myopia, ectopia lentis, glaucoma, cataract, and increased risk of retinal detachment. Skeletal system findings include disproportionately long extremities for the size of the trunk (dolichostenomelia), joint laxity, pectus excavatum or pectus carinatum, and scoliosis.

Clinical diagnosis based on the “Ghent nosology'' was revised in 2010. 2 *FBN1* (OMIM *134797) gene, located on 15q21.1, is responsible for Marfan syndrome. *FBN1* is a relatively big gene containing 65 exons and it encodes a 350 kDa glycoprotein called fibrillin. 3 Fibrillin protein controls the stability of extracellular microfibrils. If there is not a known family history of Marfan syndrome, the diagnosis should be made with:

An *FBN1* pathogenic variant known to be associated with MFS AND one of the following:

○ Aortic root enlargement ( *Z* -score ≥2.0).
○ Ectopia lentis.
  - Demonstration of aortic root enlargement ( *Z* -score ≥2.0) and ectopia lentis OR a defined combination of features throughout the body yielding a systemic score ≥7. 4

The large clinical variability and other connective tissue disorders with similar findings are confusing factors for MFS diagnosis. In the present study, three cases are presented with Marfan syndrome.

## Case Report

### Case 1

A 13-year-old male was referred to our Medical Genetics Department from the Department of Pediatrics with a prediagnosis of Marfan syndrome. He was presented with chest pain, chest deformity, and myopia. The chest pain was present for 5 years and used to last for approximately 5 minutes. There were no associated palpitations, autonomic symptoms, presyncope or syncope, and the pain was not related to respiration or food intake. When his family history was considered, his mother revealed that her mother's two brothers were tall and thin.

**Fig. 1:**
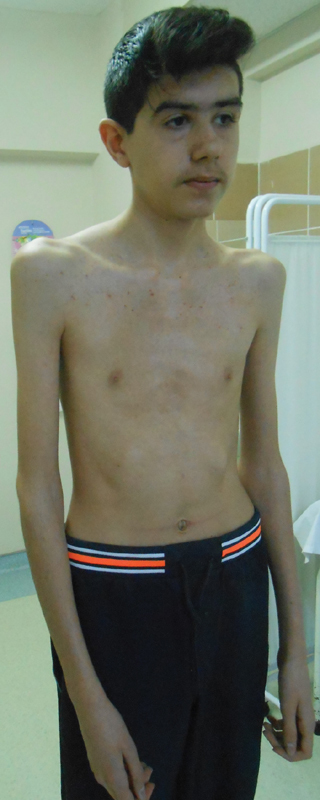
Case one with typical Marfanoid habitus.

**Fig. 2:**
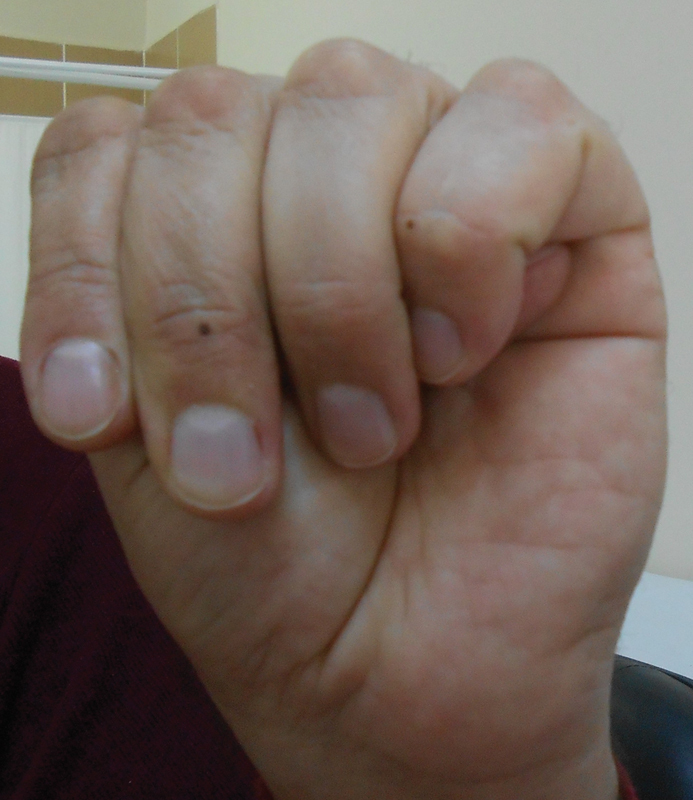
Positive thumb sign of the case of one's father.

**Fig. 3:**
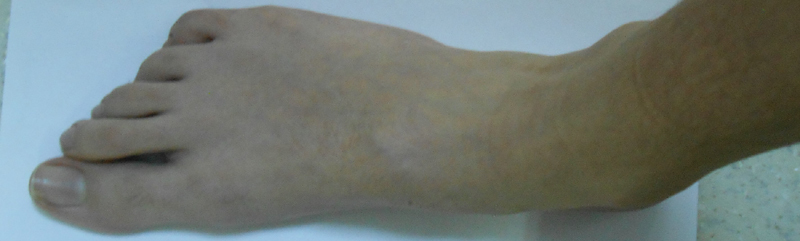
Foot of case one with arachnodactyly.

On general examination, the patient was comfortable at rest, tall (1.75 m equivalent to 97 percentile), and thin with arachnodactyly, pectus excavatum, dolichostenomelia, positive wrist, and thumb signs, increased arm span/height, dolichocephaly, scoliosis, downslanting palpebral fissures, malar hypoplasia, and keloid ( Figs. 1 and 2 ). His total systemic score was 8 based on the revised Ghent nosology. Fig. 1 demonstrates the patient's typical clinical signs of MFS. His arachnodactyly is shown in Fig. 3 .

Laboratory tests were within normal limits (full blood count, urea and electrolytes, calcium, magnesium and phosphate, and liver function tests). Echocardiography showed mitral valve prolapse, and aortic root *Z* -score was 0.23. Urinary ultrasonography showed hydronephrosis. Dynamic renal scintigraphy detected nonobstructive left kidney with a mild stasis.

Genetic evaluation of the proband revealed a heterozygous NM_000138.4( *FBN1* ):c.229G > A(p.Gly77Arg) likely pathogenic variation (PM1, PM2, PP2, PP3) on FBN-1 sequencing. After family evaluation, father and grandmother (father's mother) showed Marfan syndrome characteristics. We detected the same variation in father and father's mother.

### Case 2

A 33-year-old female patient was referred with ascending aortic aneurysm and a prediagnosis of Marfan syndrome. She had an aortic valve operation 2 years ago due to severe chest pain and nausea. There was no positive family history except her mother and father had hypertension.

Physical examination revealed a tall woman of thin habitus with arachnodactyly. She had positive wrist or thumb sign, malar hypoplasia, striae on skin, and myopia. Her total systemic score was 3 based on the revised Ghent nosology. *Z* -score was 12.26.

Laboratory tests were within normal limits (full blood count, urea and electrolytes, calcium, magnesium and phosphate, and liver function tests). Echocardiography showed functional artificial aortic valve, mild aortic regurgitation, and ascending aorta graft.

Molecular analysis for Marfan syndrome was planned for this case; the result showed pathogenic heterozygous novel NM_000138.4( *FBN1* ):c.165–2A > G variation (in-silico analysis PVS1, PM2, PP3, and PS2). As the analysis of parents showed no pathogenic variation, the case underwent de novo Marfan syndrome diagnosis.

### Case 3

Three year and 9 month-old-female patient consulted our clinical genetics department with arachnodactyly and micrognathia. Her parents were nonconsanguineous and there were no positive findings in the family history except nephrotic syndrome in her mother. She was the first child of the family and mother had preeclampsia in the prenatal period. She was born through cesarian-section in 38 weeks with a suspicious history of meconium aspiration/asphyxia. Her birthweight was 2,850 g (10–25 p) and growth parameters were mildly retarded. According to the parents she had aggression and used to forget the names of the colors.

On examining the 3-year 9-month old patient, weight came out to be 15.7 kg (75–90 p). She had a broad forehead, bilateral epicanthus, prominent nose, retromicrognathia, thin lips, pectus excavatum, and positive thumb sign. In echocardiography, she had mitral valve prolapse and mild mitral insufficiency. *Z* -score was 0.5. Molecular analysis revealed a heterozygous novel NM_000138.4( *FBN1* ):c.399delC(p.Cys134ValfsTer8) variation (in-silico analysis PVS1, PM1, PM2, PP3, and PS2). This variation is associated with Marfan syndrome. We analyzed the parents, but they both did not reveal the same variation. Our case was diagnosed as de novo Marfan syndrome.

## Molecular Analysis

After obtaining the informed consent form from the cases/families 2-mL EDTA (ethylenediaminetetraacetic acid) peripheral blood samples were obtained from the patients. Isolation of deoxyribonucleic acid (DNA) from peripheral blood samples was done using EZ1 DNA blood 200-µL isolation kits (Qiagen, Hilden, Germany) in EZ1 Advanced XL (Qiagen, Hilden, Germany) nucleic acid isolation device. DNA assay was performed with the Qubit dsDNA HS Assay Kit (Invitrogen).

Sequence analysis of 15 genes was performed using Qiaseq-Targeted DNA Panel Kit (CDHS-14630Z-997) (Illumina). *COL3A1, COL5A1, COL5A2, EFEMP2, FBN1, FBN2, NOTCH1, SKI, SLC2A10, SMAD2, SMAD3, TGFB2, TGFB3, TGFBR1, TGFBR2* genes were analyzed. The variant analysis was performed by using Qiagen Clinical Insight software.

## Discussion

MFS is a connective tissue disorder including aortic root dilatation, ocular lens dislocation, overgrowth of the long bones and chest deformity. Connective tissue disorders have a wide variability of phenotypes. MFS-related disorders have similar symptoms and thus, differential diagnosis should be done carefully. Our cases in this study were diagnosed with suspected Marfan syndrome considering revised Ghent nosology and molecular test results; the cases received the accurate diagnostic prognosis of MFS.

It has been reported that *FBN1* gene mutations are the causes of MFS. Less than 10% of the patients with typical clinical characteristics of MFS have *TGFBR* gene mutations. 5

*FBN1* is mapped to chromosome 15q21.1 and encodes a 2,871 amino acid protein. Pathogenic variations of *FBN1* may cause formation anomalies of fibrillin and microfibrils. *FBN1* is expressed in different tissues and especially cardiovascular system, cornea, and cartilage are affected with the mutations of the *FBN1* gene. 3 FBN1 protein maintains microfibers and includes transforming growth factor-1 (TGF-1) binding protein-like domains and calcium-binding epidermal growth factor-like domains (cbEGF).

Missense FBN1 mutations are generally localized in cbEGF which disrupts the stability of elastic fibers. 6 Patient one and patient two had missense mutations with MFS clinical features. Missense mutations affect the structure of fibrillin-1 and disrupt the function. 7 Thus, missense mutations are leading to disorganized microfibrils and effect the connective tissue.

TGFBR1, TGFBR2, and SMAD3 genes also affect the pathway of TGF-β, such as the *FBN1* gene. 8 If there is dysregulation of TGF-β signaling, the risk of thoracic aortic diseases increases. Cardiovascular diseases are the most significant clinical manifestations of MFS and MFS-related disorders. Cardiovascular pathologies, such as aortic rupture and aortic dissections may be life-threatening. The success of surgical management in aortic diseases states the survival of patients. 9 In addition, education of patients about the symptoms and risks is significantly important for taking care of themselves. Genetic counseling is important for these patients or other cases who have a family history. Patient two had an aortic valve replacement operation when she was 31 years old.

Mitral valve prolapse is another severe pathology in MFS. It is reported as mitral valve prolapse is found in 40 to 54% of the patients with MFS. 10 Twenty-five percent of these patients have moderate to severe mitral regurgitation.

The variation of patient one was a missense mutation with rs794728290 the database of single nucleotide polymorphisms (dbSNP) number. The predictions of this variation were, mutation taster: disease-causing, Sorting Intolerant From Tolerant (SIFT): tolerated, GERP (genomic evolutionary rate profiling) score: 5.1199, and DANN (deleterious annotation of genetic variants) score: 0.9992. The same variation was found in his father and father's mother with clinical findings of MFS. Autosomal dominant inheritance of MFS was seen in this family; the family screening is crucial in MFS. There may be different phenotypes within intrafamilial members with MFS as seen in this family. The father of this case (patient 1) had ascending aorta enlargement and left ventricular type 1 diastolic dysfunction. He had an operation for chest deformity. Tissue healing was difficult, so a graft was taken from his leg. The grandmother (mother of his father) had chest deformity and cardiac valve pathology.

It is known that it is difficult to explain this complex genotype–phenotype correlation of MFS. A novel and de novo missense variation was detected in patient two. Approximately 25% of the MFS patients have a de novo FBN1 pathogenic variant. 4 This variation is a A > G transition in splice site. The novel deletion in the *FBN1* gene observed in our patient three was associated with MFS. Single base deletion caused a stop codon in this pathogenic variation. Splice variant would possibly lead to alternative splicing of Exon 3.

Although the phenotype–genotype relationship in MFS is not fully understood, pathogenic variations are thought to predominantly affect the fusion of microfibrils. 11 There are experimental studies showing that fibrillin 1 is effective in achieving tissue homeostasis rather than elastin formation. 11 Among the mechanisms causing the disease, decreased fibrillin-1 synthesis and pathogenic variations of exons in the central region of the gene can be counted. This difference also explains the clinical spectrum, which can range from severe neonatal Marfan to isolated ectopia cordis. 12 The relationship between the various pathogenic variations and the phenotype is insufficient and the clinical setting determines other risk factors specific to the patient. The only exception to the weak correlation between genotype and phenotype is neonatal MFS with a fatal course.

As *FBN1* is a great gene, analyzing it with next-generation sequencing gives accurate and cost-effective results in a short time. In this study, we analyzed 15 genes ( *COL3A1, COL5A1, COL5A2, EFEMP2, FBN1, FBN2, NOTCH1, SKI, SLC2A10, SMAD2, SMAD3, TGFB2, TGFB3, TGFBR1, TGFBR2)* to detect Marfan syndrome and other associated disorders. A patient associated with arachnodactyly may be suspected with Marfan syndrome; after molecular analysis only FBN2 variation may be detected in this patient and the diagnosis will be changed. Both for cost-effectiveness and differential diagnosis, studying gene panels is preferable.

## Conclusion

Our study added novel mutations of *FBN1* gene to MFS clinical characteristics to the genotype–phenotype spectrum for the literature. Molecular analysis has an important role in the accurate diagnosis of MFS and MFS-related disorders. Correlation of clinical findings and molecular analysis will be helpful for genetic counselling, prenatal diagnosis, and management of patients with the same variations on the prognosis prediction.
